# From Data to Truth in Psychological Science. A Personal Perspective.

**DOI:** 10.3389/fpsyg.2017.00702

**Published:** 2017-05-16

**Authors:** Fritz Strack

**Affiliations:** Department of Psychology, University of WürzburgWürzburg, Germany

**Keywords:** replication, social psychology, methodology, critical discourse, theory testing

## A failure to replicate

Over 30 years ago, Leonard Martin, Sabine Stepper, and I (Strack et al., 1988) conducted two studies to test the “facial feedback” hypothesis (Darwin, 1872). At the time, the hypothesis itself, namely that facial expressions may affect emotional experiences, was well established and frequently tested (e.g., Leventhal and Mace, 1970; Laird, 1974). However, the underlying mechanism remained largely unexplored. On the one hand, the feedback could have been mediated by an inference from the emotional meaning of the expression to the underlying emotional state. On the other hand it could have been a more direct mechanism that does not involve any inferences. To resolve this ambiguity, we attempted to eliminate the inferential route by preventing participants from interpreting their facial action as “a smile.” This was accomplished by having participants holding a pen either between their protruded lips, which prevents smiling, or between their teeth, which facilitates it. Holding the pen in either of these positions, participants had to perform a series of tasks, among them a rating of the funniness of cartoons. As predicted, the cartoons were rated to be funnier if the pen was held between the teeth than between the lips. The effect was not strong but met the standard criteria of significance.

The resulting “pen study” was meant neither to demonstrate a cute phenomenon nor to identify a powerful intervention to improve people's feelings. Instead, it was intended to be one piece in the theoretical puzzle. This was underscored in the last sentence of the original article: “Obviously, more research is needed to understand the exact mechanisms that are responsible for facial feedback. In this endeavor, an alternative methodology that eliminates possible confounds may be helpful (p. 776).”

Since 1988, more research on facial feedback has been conducted (Laird and Lacasse, 2014) and even a new methodology was introduced that was robust enough to even afford therapeutic interventions, namely the suppression (injecting Botox) of the corrugator muscle, which contracts the eyebrows and is implied in negative emotions (Finzi and Rosenthal, 2016).

In 2013, I volunteered to be part of a Registered Replication Report project and submitted the experimental materials. In September 2016, the results came out (Wagenmakers et al., 2016). Seventeen replication groups had tested 1,894 participants. For nine groups, the result was in the predicted direction, for eight groups in the opposite direction. Overall, there was no significant effect.

## What have we learned?

Based on this outcome, the question arises: What have we learned? More generally stated: What can be learned from non-replications? There are three possibilities. First, the original finding was the result of fraud or cheating. Second, the original finding was not “real,” not a “true” effect. Third, the original effect was weak and fragile, not robust enough to show up under changing conditions.

### Fraud

This happens in science and psychology is no exception. But although non-replications are no convincing proofs of data fabrication, they always suggest this possibility between the lines. For any original authors, this is a bit of a no win situation, because protesting too much may increase suspicion. Therefore, if anyone believes that fraud has taken place, they should explicitly state so. Vague insinuations of p-hacking or questionable research practices are inappropriate.

### Not “real,” not “true”

As a second possibility, it is claimed that a failed replication shows that the original finding was not a “real,” or not a “true” effect. Here, things become even more complicated because unlike propositions, effects have no truth values. Correctly, one could claim that the proposition describing the original outcome is false because an error has occurred. Then, this error, e.g., a confounding influence, should be identified. However, when replicators talk about truth, they do not adopt the logic of propositional veracity but a probabilistic theory, namely the theory of Null Hypothesis Significance Testing and identify a Type I error, which is the incorrect rejection of a true null hypothesis (a “false positive”) when the original effect was produced by chance.

Unfortunately, we are deeply entrenched in this statistical terminology and fail to ask about the causal determination of a “false” positive. Let us assume we are rolling some dice and come to the conclusion that resulting numbers are produced by chance. This conclusion does not imply that the laws of mechanics have not been operating. It just means that the interaction of the various influences has been so complex that it was not possible to generate a prediction. But in principle, a chance outcome is just as causally determined as any other mechanical phenomenon.

Applied to the current replication results, one might ask about the determinants that caused nine teams to replicate the original findings and eight teams to obtain results in the opposite direction. Participants' prior knowledge might be one possibility (Strack, 2016). However, adherents of the theory of Null Hypothesis Significance Testing seem to assume that chance is causally undetermined. But if it is the task of a scientist to identify the causes of things, it seems highly problematic to assume that some effects are “false” in the sense of being causally undetermined. In particular, as the major prerequisite of the underlying statistical theory, namely the existence of a finite population from which a random sample is being drawn, is notoriously violated.

Finally, a non-replication may suggest that the effect is *subtle and fragile*. At first sight, such a weakness seems to be a constant characteristic of the independent variable. However, as effect sizes are not determined in a universe that is purified of all other influences, observed strength is determined by both the systematic variance between and the error within the experimental conditions. In other words, irrelevant factors (e.g., the context, person characteristics) determine the strength of an effect. As a consequence, its size is not a constant characteristic but is codetermined by the contextual and personal influences. Moreover, its size may vary over time. It may be weak at the beginning of a research program where little is known about the relevant conditions, but may increase as the relevant conditions become known and controllable.

Of course, the strengths of psychological interventions may vary. In the case of facial influences, the impact of the zygomaticus muscle on emotional experiences seems to be less robust than that of the corrugator (Finzi and Rosenthal, 2016). In the domain of social judgments, the anchoring effect (Strack and Mussweiler, 1997; Strack et al., 2016) seems to be particularly strong. But what follows from that?

There is no doubt that the strength of an effect matters if an experimental procedure is evaluated to be suitable as an intervention in natural, applied settings, where it is less important to understand the causes of an effect than to assure its efficiency, like tests of drugs in clinical trials. Thus, the pen procedure may not be effective to alter moods in natural settings. However, when it comes to basic research, the strength of an effect is much less relevant because basic science is not about demonstrating or generalizing effects but about testing theories. And if one procedure turns out to be too fragile, basic scientists may try to reduce the noise or to find a more robust equivalent.

## Insights

Authors of a study that has become a target of a replication exercise face a particular challenge. If their findings are replicated, they are relieved and get off the hook. If not, they are expected to accept the verdict of the tribunal and promise to do better in the future. Although, the situation is not satisfying, they may arrive at the insight that there exists no direct route from data to truth. Instead, they may come to the conclusion that science is about arguments that should be based on empirical evidence whose validity, however, is not merely determined by probabilistic parameters. Although, power, effect size, significance level, etc. provide useful information, they deliver no immediate link to the truth or falsehood of a hypothesis. Instead, they must be critically evaluated (Popper, 1959), not only by statisticians but by scientists who are experts in the field. For such communicative exchanges to be effective, participants must be informative (Grice, 1975).

In the current debate, this prerequisite has often been overlooked. For example, it has frequently been deplored that journals have a confirmation bias such that negative results are rarely published. As a remedy, it has been proposed to preregister the procedures of a study with the editors to assure that the results will be published regardless of their results. Although, this seems impartial, such a publication may not be informative and journals run the risk of becoming mere archives instead of media of the debate. As a consequence, it is not surprising that most journal editors prefer positive outcomes that add something new to what is already known.

In contrast, what is informative for replications? Not that the original finding has been replicated, but that it has been “overturned.” Even if the editors' bias (Gertler, 2016) bias is controlled by preregistration, overturned findings are more likely to attract readers' attention and to get cited. In a scientific debate, both tendencies contribute to a critical evaluation that may create new insights.

However, there is an important difference between these two biases in that a positive effect can only be obtained by increasing the systematic variance and/or decreasing the error variance. Typically, this requires experience with the subject matter and some effort in controlling unwanted influences, while this may also create some undesired biases. In contrast, to overturn the original result, it is sufficient to decrease the systematic variance and to increase the error. In other words, it is easier to be successful at non-replications while it takes expertise and diligence to generate a new result in a reliable fashion. If this is the case, it should be reflected in measures of academic achievement, e.g., in the h-index or in the number of previous publications. Although, the last word is not yet spoken, data from Gertler (2016) and Bench et al. (2017) suggest that this asymmetry may be empirically founded.

## Future perspectives

As things stand now, I am not optimistic about the impact of the Registered Replication Reports on the field. Strong effects will be replicated, weak effects not. If this incentivizes researchers to pursue “strong” effects rather than theoretically informative ones, it may shift the field into a more applied direction and away from theoretical innovation. And as long as the outcomes are not embedded in a critical debate, they are seen as final verdicts on an “effect” without a clear message on any underlying process. Moreover, non-replications may spread doubt about the integrity of the original research while the public discussion about the percentage of studies that cannot be replicated does not add to the reputation of our field (e.g., Johnson et al., 2017).

In their introduction to the 2016 volume of the Annual Review of Psychology, Susan Fiske, Dan Schacter, and Shelley Taylor point out that a replication failure is not a scientific problem but an opportunity to find limiting conditions and contextual effects. To allow non-replications to regain this constructive role, they must come with conclusions that enter and stimulate a critical debate. It is even better if replication studies are endowed with a hypothesis that relates to the state of the scientific discourse. To show that an effect occurs only under one but not under another condition is more informative than simply demonstrating non-effects (Stroebe and Strack, 2014). But this may require expertise and effort.

If there is a crisis of psychology, it is an epistemological crisis restricting its discourse to a probabilistic model that promises a direct path from data to truth. Under this perspective, psychology may deteriorate to a collection of effects and phenomena that mainly differ in their strength (e.g., Yap et al., 2017). It is no longer a joint exploration of the basic laws of human behavior. Science progresses through critical discourse, and this is what must be revived again.

## Author contributions

The author confirms being the sole contributor of this work and approved it for publication.

### Conflict of interest statement

The author declares that the research was conducted in the absence of any commercial or financial relationships that could be construed as a potential conflict of interest.
